# Eyes Closing and Drowsiness in Human Subjects Decrease Baseline Galvanic Skin Response and Active Palmar Sweating: Relationship Between Galvanic Skin and Palmar Perspiration Responses

**DOI:** 10.3389/fphys.2020.558047

**Published:** 2020-12-10

**Authors:** Hideya Momose, Norimasa Morimitsu, Eiji Ikeda, Shigeki Kanai, Masao Sakaguchi, Toshio Ohhashi

**Affiliations:** Department of Innovation of Medical and Health Sciences Research, Shinshu University School of Medicine, Matsumoto, Japan

**Keywords:** GSR, perspiration ratemeter, palmar sweating, eyes closing, drowsiness, amygdala

## Abstract

We previously constructed a perspiration ratemeter for the measurement of palmar sweating in human subjects. Although galvanic skin response (GSR) has been used to evaluate emotional responses in human subjects, little is known about the relationships between the phasic and baseline components in GSR and active palmar sweating. From the aforementioned, we aimed to investigate the relationships in human subjects with handgrip exercise and eyes closing or opening. Fifteen healthy volunteers (mean age: 26.9 ± 8.7 years) participated in the present experiments. We investigated the effects of maximal handgrip exercise, eyes closing or opening, and self-awareness of drowsy on the GSR, active palmar sweating, R-R interval in electrocardiograph (ECG), and percentage of α wave in EEG. The faster phasic component in GSR completely agreed with the starting point of active palmar sweating. Handgrip exercise induced significantly faster spike in GSR, active palmar sweating, and decrease in R-R interval in ECG. Eyes closing produced significant decreases in baseline GSR and active palmar sweating in all human subjects. The percentage of α wave in electroencephalograph (EEG) also increased. In contrast, eyes opening increased significantly the baseline GSR and active palmar sweating. In the equivalent electrical model of human skin, the eyes closing–mediated time-dependent decrease in the baseline GSR completely agreed with the hypothesis that the palmar skin voltage only in the model decreased time dependently to 0.4 of the control during 6 min. The self-awareness of drowsy in mid-night working with computer produced similar decreases in baseline GSR and active palmar sweating to the responses with eyes closing in all human subjects. In conclusion, the faster spike in GSR completely agreed with the starting point of active palmar sweating. Eyes closing and opening or self-awareness of drowsy significantly produced changes in baseline GSR and active palmar sweating, which may become useful tools for evaluating clearness or drowsiness in human subjects.

## Introduction

Previously, we designed and constructed a high-sensitivity perspiration ratemeter using capacitive humidity sensors which is applicable to the direct measurement of palmar sweating in human subjects (Ohhashi et al., 1998). The safety of the ratemeter has been approved by the Japanese Government (approval no. 21600BZZ00433000, 2004, 2017) and subsequently officially accepted for use with patients in hospitals. Using the ratemeter, active palmar sweating was confirmed to collaborate with neural activities of the limbic-cortical centers including the amygdala, hippocampus, and prefrontal cortex (Homma et al., 1998, 2001). The sudomotor pathways from these centers run through the brain stem, spinal cord, and peripheral cholinergic sympathetic nerve fibers to the eccrine glands in palmar skin (Homma et al., 1998, 2001). In fact, non-selective viral encephalitis in the amygdala in young patients did not induce active palmar sweating, although physiological palmar sweating was detected approximately 2 weeks after drug treatment (Asahina et al., 2003).

In contrast, galvanic skin response (GSR) is well known to reflect changes in the electrical properties between palm and forearm skin in human subjects, which are produced by noxious and emotional stimulation, and aroused alertness (Darrow, 1927, 1937; Edelberg, 1977; Fowles, 1986; Denda et al., 2001; Boucsein, 2012). The stimulation-mediated phasic component of GSR agreed with the increase in electrical conductance of the skin (a decrease in resistance) between palm and forearm in human subjects (Darrow, 1927, 1937; Edelberg, 1977; Fowles, 1986; Denda et al., 2001: Boucsein, 2012). The changes in GSR are also produced by the excitation of cholinergic sudomotor nerves (Darrow, 1937; Edelberg, 1977; Fowles, 1986). The GSR signals are considered to result from two additive processes, consisting of a tonic baseline level driver, which fluctuates very slowly, and a faster phasic component. Psychology researchers assessing GSR have mainly focused on the latency and amplitude of the phasic spikes (Newman and Blanton, 1970; Roy et al., 1993). However, the mechanisms of changes in tonic baseline levels of GSR still remain unsolved.

From the aforementioned, we aimed to (1) first clarify the relationships between the components in GSR and active palmar sweating using maximal handgrip exercise, and fluctuations in the R-R interval in electrocardiograph (ECG) recordings, and (2) observe active palmar sweating with handgrip exercise using a microscope. (3) Next, we investigated the effects of eyes closing and opening in human subjects according to self-control condition on the changes in tonic baseline GSR and palmar sweating, alongside simultaneous measurement of the percentage of α wave electroencephalograph (EEG) recordings. (4) We also evaluated the effects of self-awareness of drowsy during the operation of computer at mid-night on the changes in GSR, percentage of α wave in EEG, and R-R interval in ECG. In addition, to confirm the drowsiness of volunteers, research assistants checked the number of eyes closing and head down in the volunteers during the computer working behind a transparent wall. Finally, (5) we simulated the changes in the baseline GSR using an equivalent electrical model of human skin corresponding to the physiological and morphological properties of palmar and forearm epidermis in human subjects (Kuno, 1934, 1956). Table 1 shows the different experimental protocols.

**TABLE 1:** The experimental protocols.

**The experimental protocols**
1. Observation with a microscope for active palmar sweating in human subjects with maximal handgrip exercise (*n* = 15).
2. Effects of maximal handgrip exercise on GSR, active palmar sweating, and R-R interval in ECG in human subjects were examined (*n* = 15).
3. Effects of eyes closing and opening on GSR, active palmar sweating, R-R interval in ECG, and percentage of α wave in EEG in human subjects were investigated (*n* = 15).
4. Effects of self-awareness of drowsy on GSR, active palmar sweating, R-R interval in ECG, and percentage of α wave in EEG in human subjects were evaluated (*n* = 15).
5. Simulation with equivalent electrical model of human skin for eyes closing and opening-mediated changes in the baseline GSR was conducted.

## Materials and Methods

### Subjects

In total, 15 healthy volunteers (mean age: 26.9 ± 8.7 years, 8 males, 7 females) without any diseases participated in the present experiments. The minimum number of volunteers was recommended by the ethical committee for clinical observation study. Hence, the number of participating volunteers was decided to be suitable for obtaining the conclusion. We selected the younger subjects because the older persons more than 50 years old have known to become less responsive to palmar sweating (Ohhashi et al., 1998). The ethical committee for human clinical studies at the School of Medicine, Shinshu University, approved the study (approval no. 4445 on August 6, 2019). All subjects provided their written and oral informed consent. All data and procedures conformed to the tenets of the Declaration of Helsinki. The human experiments were conducted in the afternoon ranging between 13:00 and 16:00 pm, considering the circadian rhythm of the human autonomic nervous system. The temperature and moisture of the examination room were maintained at 22–23°C and 40–50% using air conditioners, respectively. We also took care to keep silent in the experimental room to minimize emotional stress for the volunteers. Before starting the experiments, all subjects washed with soap to clean up their hands and forearms. The subjects were prohibited water and food intake, and urine excretion from 1 h before the experiments.

### Measurement of Active Palmar Sweating With the Perspiration Ratemeter

We previously designed and constructed a new perspiration ratemeter to directly measure skin perspiration on human subjects. We have demonstrated the properties of perspiration ratemeter in detail (Ohhashi et al., 1998). Active palmar sweating in all volunteers was measured using the perspiration ratemeter (SKN 2000; SKINOS, Nagano, Japan) attached to the surface of palmar skin (contacted area 1 cm^2^). The amount of active palmar sweating was demonstrated as water loss per constant area and time using a calibration curve. With the step response of the ratemeter, the time to obtain a maximal response is ∼1.5 s. The changes in environmental temperature ranging from 15 to 35°C, and environmental humidity ranging from 30 to 70%, do not affect the output of the ratemeter. The correlation between water loss in active palmar sweating and changes in output voltage of the ratemeter is approximately linear, r^2^ = 0.983. The sensitivity of the electrical performance is 0.1 V/1 mg water loss/5 min.

### Monitoring of Active Palmar Sweating With a Microscope

We designed and constructed an apparatus capable of directly observing active sweating on the palmar skin alongside quantitative measurement of active sweating (Kamei et al., 1997). The apparatus was composed of a microscope equipped with a CCD camera (magnification, ×100–×1,000, Model 6110; Keyence, Osaka, Japan), an XYZ stage, a VHS video cassette recorder (HV-F92; Mitsubishi, Tokyo, Japan), a video timer (VTG-33; For A, Tokyo, Japan), and the perspiration ratemeter. For obtaining quantitative data for observation of active palmar sweating, the appeared time of sweat on the palmar skin in human subjects with maximal handgrip exercise was measured. Simultaneously, the absorbed time of the sweat into the palmar skin was also measured.

### Measurement of Galvanic Skin Response With Ag/AgCl Skin-Surface Electrodes

**FIGURE 1:**
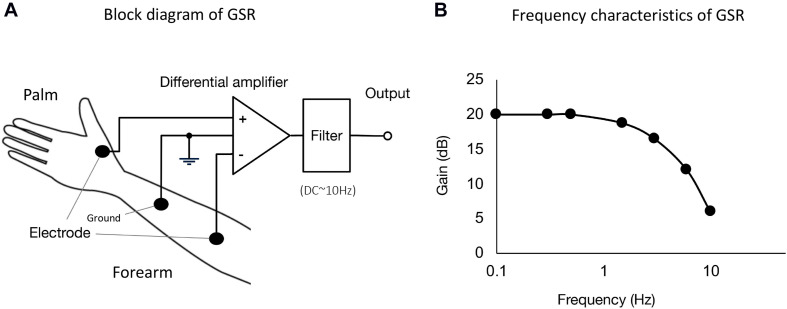
**(A)** Block diagram of GSR used in the present experiments. We designed and constructed GSR with Ag/AgCl disposable electrodes and an adhesive gel (Nihon Koden, Tokyo, Japan). A differential amplifier (INA118; Texas Instruments, Dallas, TX, United States) with a filter was used to measure changes in electrical voltage difference between the two electrodes, which were attached on the skins at the bottom of the thumb and forearm skin near the cubital joint. The ground electrode was fixed at the middle position of the skin surface between palm and forearm. The output signal is low-pass filtered at 1 Hz. **(B)** Frequency characteristics of the GSR. The GSR output is measurable for voltage changes ranging from DC to ∼1 Hz.

We designed and constructed the GSR system with Ag/AgCl disposable electrodes and adhesive gel (Nihon Koden, Tokyo, Japan), especially suitable for recording voltage changes in the baseline GSR. Figure 1A shows a block diagram of the GSR system. A differential amplifier (INA118; Texas Instruments, Dallas, Texas, United States) with a filter was used to measure changes in electrical voltage between the two electrodes, which were attached to the skins of the palm at the bottom of the thumb and forearm near the cubital joint. The ground electrode was fixed around the middle position of skin surface between the recording electrodes. Thus, to evaluate electrical voltage changes in GSR, the constant voltage is offered to the differential amplifier. The output signal is low-pass filtered at 1 Hz. The frequency characteristics of the constructed GSR device is shown in Figure 1B.

### Measurement of Changes in R-R Interval With ECG Recording

To evaluate the differences of neuronal activities of autonomic nerve fibers innervated on heart and the sudomotor nerve fibers in sweat glands in human subjects, the changes in R-R interval in ECG were evaluated (SKINOS MSD-001; SKINOS, Nagano, Japan) with a heart rate monitor (AD8232; Analog Devices, Norwood, MA, United States). The recording and ground electrodes of the ECG were fixed on the skin surface at thorax and upper extremity of human subjects, respectively.

### Measurement of Changes in Percentage of α Wave by Electroencephalography Recording

To evaluate the neuronal activity of the central nervous system (CNS) in human subjects, changes in the percentage of α wave in EEG were measured with a portable EEG (Muse Brain System; Digital-medic, Kyoto, Japan) equipped with a computer and software (Digital-medic, Kyoto, Japan).

### Experimental Protocols

All subjects were seated on comfortable chairs with back support and then relaxed in a silent air-conditioned room. All subjects were equipped with the ECG and EEG electrodes, placed on their skin surface of their thorax, forearm, and head. Their palm and forearm skins were also fixed with the Ag/AgCl electrodes for GSR measurement. The probe of the perspiration ratemeter was placed on the palmar skin at the bottom of the thumb at the same hand. First, the subject performed the 5-s handgrip exercise at 100% maximal voluntary contractions 4–5 times with 60-s interval to confirm the reproducible palmar sweating responses. The handgrip exercise with these parameters is known to produce no or little adaption in active palmar sweating (Kobayashi et al., 2003; Tomioka et al., 2005). In addition, the correlation between the GSR output and the water loss of palmar sweating was evaluated with 5 s maximal handgrip stimulation with two times in all human subjects (*n* = 30). The R-R interval in ECG was simultaneously recorded with maximal handgrip exercise. Next, all subjects performed the eyes closing and opening with each self-control condition because of reducing emotional stress by the order of research assistants. The eyes closing and opening in the volunteers were also confirmed by the research assistants seated behind a transparent wall.

Next, to evaluate the self-awareness of drowsy in human subjects, we recorded changes in the baseline GSR and active palmar sweating with simultaneous recordings of EEG and ECG when the 15 volunteers operated a computer at mid-night between 22:00 and 00:00. Simultaneously, the research assistants checked the numbers of the eyes closing and head down in the volunteers during the computer working behind transparent walls. After the experiments, the research assistants demonstrated the obtained data and the numbers of the eyes closing and head down measured simultaneously by the research assistants to all volunteers. The volunteers confirmed by themselves the self-awareness of drowsy with considering the changes in the all data.

### Simulation

To clarify mechanisms of the eyes closing and opening-mediated changes in the baseline GSR, we simulated changes in baseline GSR level using the equivalent electrical model of human skin combined with morpho- and physiological properties of human palm and forearm skin; the thickness of epidermis, the length of duct of sweat gland, physiological function of eccrine sweat gland, i.e., reabsorption and secretion properties of water and electrolytes, especially sodium, chloride, and potassium ions (Kuno, 1934, 1956; Denda and Tsuchiya, 2000; Denda et al., 2000; Grimnes and Martinsen, 2003).

### Statistical Analysis

All GSR data are represented as the mean ± SEM. Statistical significance was analyzed using Student’s *t*-test for unpaired observations after checking that the data were suitable for a *t*-test distribution or a one-way ANOVA followed by a Duncan’s post hoc test, as appropriate. *p* < 0.05 was considered statistically significant. The associations between GSR output and water loss in palmar sweating, and between time delay in GSR and in active palmar sweating were compared by linear regression. Pearson’ ratio, *r*^2^ was obtained.

## Results

### Observation for Active Palmar Sweating in Human Subjects With a Microscope

**FIGURE 2:**
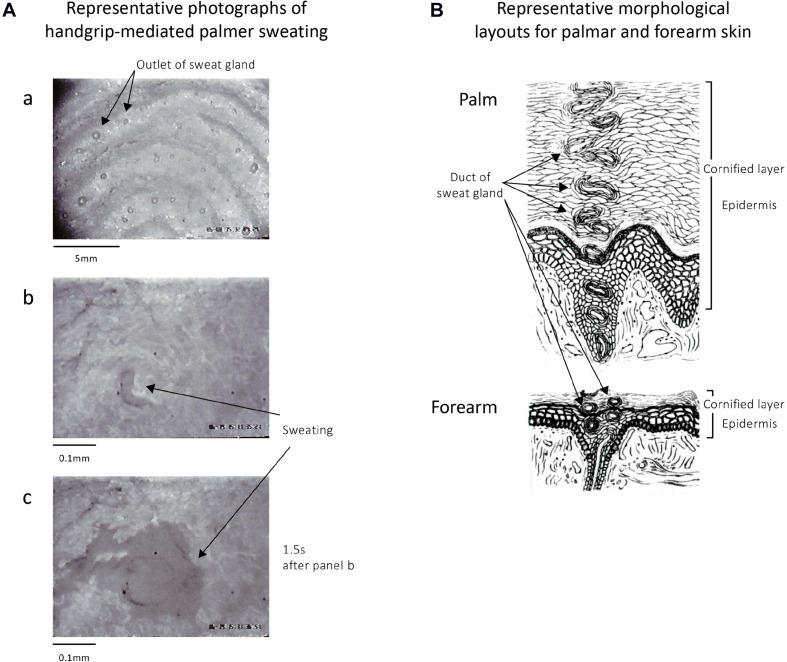
**(A)** Representative photomicrographs of skin surface of thumb in a human subject before and after active palmar sweating. **(A-a)** The sweat gland outlets are found on the top of the finger print of the thumb. **(A-b)** Immediately after maximal handgrip exercise–mediated palmar sweating in the same subject. The black shadow shows the sweat. **(A-c)** A total of 1.5 s after starting the handgrip-mediated palmar sweating in the same subject. The sweat shown by black shadow diffused rapidly on the surface of surface skin of thumb and then was quickly absorbed into the epidermis of the skin after several seconds. **(B)** Representative layouts of the palmar and forearm skin in human subjects summarized the data written in classical books (Kuno, 1934, 1956). The thicker epidermis and longer duct of the sweat gland in palmar skin are characteristic morphological properties compared with those in the forearm skin.

Figure 2A demonstrates representative microphotographs observed before and after active palmar sweating in a human subject with maximal handgrip exercise. As shown in Figure 2A-a, the outlets of the sweat gland on the surface of thumb were observed with nearly constant distance at the top region of finger prints. Immediately after starting maximal handgrip exercise, a small sweat shadow was observed at the outlet of sweat gland (Figure 2A-b). At 1.5 s after starting active sweating, the shadow of sweat diffused rapidly on the surface of the thumb (Figure 2B-c), and then was quickly absorbed into the cornified layer of epidermis in the palm. Table 2 shows quantitative data. Thus, the appeared time of the sweat after starting maximal handgrip exercise was 1.43 ± 0.15 s (*n* = 30). The absorbed time of the sweat into the palmar skin was 6.02 ± 2.19 s (*n* = 30).

**TABLE 2:** The exercise-mediated changes in GSR and water loss in palmar sweating.

**Number of sample**	**Water loss in palmer sweating (mg/min)**	**Changes in GSR (mV)**
1	0.39	7.6
2	0.17	3.8
3	0.49	7.0
4	0.62	10.3
5	0.80	12.3
6	0.58	9.2
7	0.56	8.0
8	0.51	7.2
9	0.06	5.0
10	0.09	4.2
11	0.87	12.2
12	0.57	11.2
13	0.60	11.0
14	0.11	1.6
15	0.53	9.6
16	0.04	2.4
17	0.07	2.6
18	0.58	9.4
19	0.48	6.4
20	0.52	8.0
21	0.34	6.8
22	0.06	1.6
23	0.40	7.2
24	0.08	1.6
25	0.43	8.4
26	0.21	4.8
27	0.28	6.2
28	0.33	6.6
29	0.10	2.8
30	0.53	8.4
Mean	0.38	6.8
±SE	0.04	0.58

### Morphological Characteristics of Palmar and Forearm Skin in Human Subjects

Figure 2B shows the representative morphological layouts of palmar and forearm skin in human subjects summarized with classical studies (Kuno, 1934, 1956). As shown in the layouts, the epidermis in the palm is significantly thicker than that in the forearm. Thus, the length of the duct of sweat gland within the palmar epidermis is significantly longer comparing with forearm skin. In addition, no skin hair is observed in the palm. In contrast, large numbers of skin hair are found on the surface of the forearm skin.

### Relationship Between GSR and Active Palmar Sweating

**FIGURE 3:**
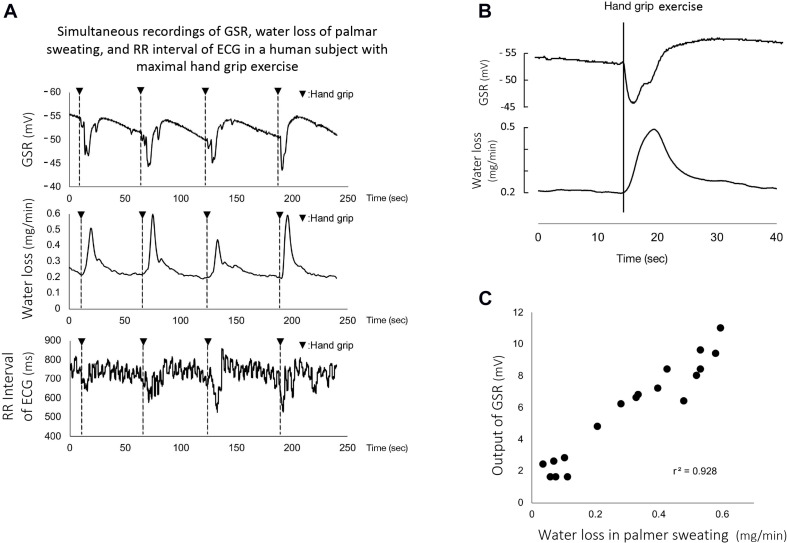
**(A)** Representative simultaneous recordings of GSR, palmar sweating, and R-R interval in ECG in a human subject during repeated maximal handgrip exercises. The water loss in the panel represents the amount of active palmar sweating calculated with the calibration curve (Ohhashi et al., 1998). The correlation relationship between the GSR output (mV) and water loss (mg/min) in active palmar sweating undergoing maximal handgrip exercise was confirmed to be approximately linear (*r*^2^ = 0.94, *n* = 30). **(B)** Representative magnified recordings of maximal handgrip-mediated GSR and active palmar sweating. Time scale shown as second scale. The starting points of the spike component in GSR and active palmar sweating completely coincided in the same subject during maximal handgrip exercise. **(C)** The correlation between the time delay in GSR and the time delay in palmar sweating (*r*^2^ = 0.75, *n* = 30). The time delay in GSR is defined as the time (s) measured between the starting points of handgrip exercise and GSR. The time delay in palmar sweating is shown as the time (s) measured between the starting points of handgrip exercise and palmar sweating, –1.5 s included with the time-delay electrical properties of the ratemeter.

Figure 3A shows representative simultaneous recordings of GSR, water loss in active palmar sweating, and changes in the R-R interval in ECG in a human subject with maximal handgrip exercise. The exercise induced reproducible faster spikes of GSR. Consistent with the GSR, active palmar sweating appeared with a short time lag. The R-R interval in ECG rapidly decreased with the changes in GSR and active palmar sweating. The correlation relationship between the GSR output and water loss in active palmar sweating was approximately linear (*r*^2^ = 0.94, *n* = 30; Figure 3B). The 30 absolute values of changes in GSR and water loss in palmar sweating are shown in Table 3 (changes in GSR 6.8 ± 0.58 mV, *n* = 30; water loss in palmar sweating 0.38 ± 0.04 mg/min, *n* = 30).

**TABLE 3:** The absolute values for the difference of time delay between the starting points for GSR and active palmar sweating.

**Number of sample**	**Delay in GSR (s)**	**Delay in water loss palmer sweating (s)**
1	0.5	0.9
2	0.7	1.7
3	0.7	1.5
4	0.7	1.3
5	0.7	1.1
6	0.7	1.4
7	0.7	1.5
8	0.6	1.0
9	0.7	1.5
10	0.6	0.9
11	0.6	1.5
12	0.8	1.5
13	0.9	1.7
14	0.5	1.0
15	0.7	1.6
16	0.5	1.2
17	0.5	1.2
18	0.6	1.3
19	0.4	1.1
20	0.4	0.9
21	0.4	1.1
22	0.6	1.5
23	0.5	1.4
24	0.5	1.2
25	0.7	1.6
26	0.5	1.3
27	0.5	1.6
28	0.5	1.5
29	0.4	1.3
30	0.5	1.2
Mean	0.6	1.3
±SE	0.02	0.04

When the electrical time-delay properties (1.5 s) incorporated with the perspiration ratemeter were corrected by the equipped computer, the starting points for the handgrip exercise-mediated GSR and active palmar sweating were entirely consistent (Figure 3C). The time delay in GSR is defined as the time (s) measured between the starting points of handgrip exercise and GSR (0.6 ± 0.02 s, *n* = 30). The corrected (1.5 s) time delay in active palmar sweating is shown as the time (s) measured between the starting points of handgrip exercise and palmar sweating (1.3 ± 0.04 s, *n* = 30), being minus 1.5 s. Figure 3D shows the summarized data for the difference of time delay between the starting points for GSR and active palmar sweating. The relationship between the time delay in GSR and the corrected time delay in palmar sweating was approximately linear (*r*^2^ = 0.75, *n* = 30). The 30 absolute values are shown in Table 3 (the delay in GSR, 0.6 ± 0.02 s; the delay in water loss, 1.3 ± 0.04 s).

### Eyes Closing and Opening in Human Subjects Produced Significant Changes in the Baseline GSR

**FIGURE 4:**
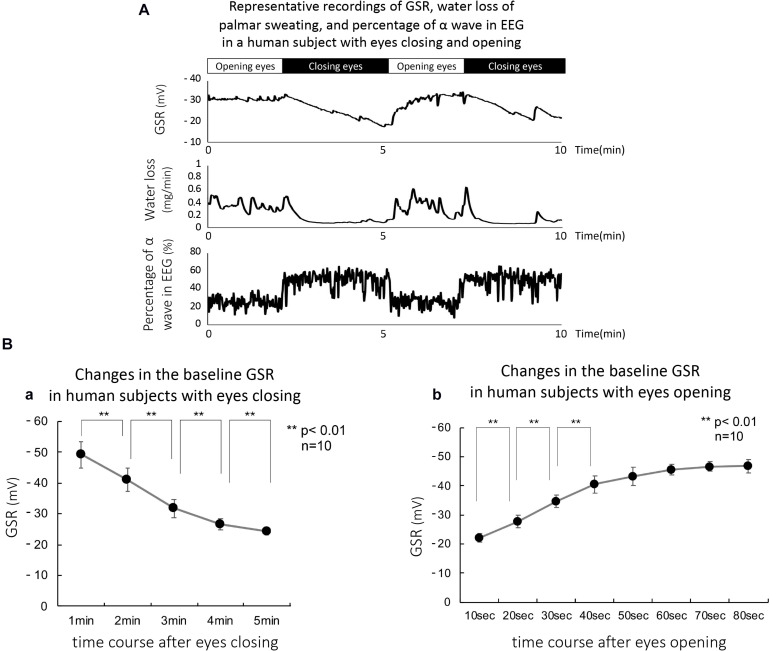
**(A)** Representative simultaneous recordings of GSR, palmar sweating, and percentage of α wave in EEG in a human subject with eyes closing and opening. **(B-a)** Summarized data for relative changes in the baseline level of GSR in human subjects with eyes closing (*n* = 15). **p < 0.01. **(B-b)** Summarized data for relative changes in the baseline GSR in human subjects with eyes opening (*n* = 15). **p < 0.01.

Figure 4A shows representative recordings for the effects of eyes closing and opening in a human subject, on GSR, active palmar sweating, and percentage of α wave in EEG. Closing eyes caused a time-dependent decrease in the baseline GSR and disappearance of active palmar sweating. Simultaneously, the percentage of α wave in EEG increased quickly. In contrast, the opening eyes induced rapid increases in the baseline GSR and appearance of palmar sweating. The percentage of α wave in EEG was decreased simultaneously.

Figure 4B-a shows the summarized data obtained with eyes closing–mediated changes in baseline GSR. Closing eyes in all human subjects produced a significant and time-dependent decrease in the baseline GSR. Approximately 5 min after eyes closing induced maximal decrease in baseline GSR (control 0 min, 1.00 ± 0.11; 1 min 0.76 ± 0.19; 2 min 0.58 ± 0.18; 3 min 0.49 ± 0.14; 4 min 0.44 ± 0.13; 5 min 0.42 ± 0.12, *n* = 15, *p* < 0.01 each value vs. control). In contrast, Figure 4B-b demonstrates summarized data obtained with eyes opening–mediated increases in baseline GSR. Approximately 50 s after eyes opening, the baseline GSR returned approximately to the levels before the starting point of eyes closing in all human subjects (control 0 s, 0.47 ± 0.11; 10 s 0.59 ± 0.15; 20 s 0.79 ± 0.13; 30 s 0.82 ± 0.15; 40 s 0.90 ± 0.14; 50 s 0.93 ± 0.14, *n* = 15, *p* < 0.01 vs. each value vs. control).

### Simulation for the Eyes Closing–Mediated Decrease in the Baseline GSR Using Equivalent Electrical Model for Human Skin

**FIGURE 5:**
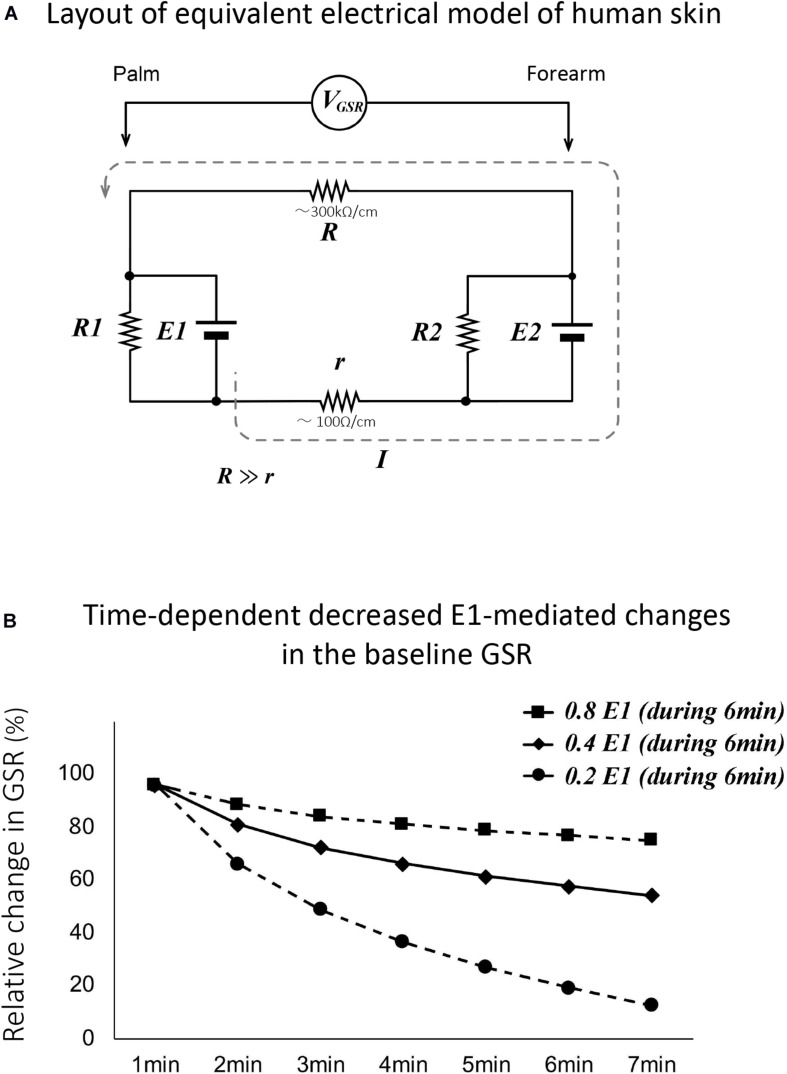
**(A)** Layout of equivalent electrical model of human skin. The voltage and resistance of palmar and forearm skin in the model are described in E1 and E2, and R1 and R2, respectively. The skin surface and the extracellular tissue resistance in palmar and forearm skin show R and r, respectively. R is known to be significantly larger than r (Denda et al., 2001). V_GSR_ shows the voltage differences between palm and forearm skin. The dotted line shows the direction of electrical current that flowed from the extracellular tissues to skin surface between palm and forearm. Thus, the skin surface electrical voltage in palm is negative. **(B)** Simulated data for changes in the baseline GSR in human subjects with eyes closing with hypothesis that the electrical voltage in palmar skin (E1) only decreased time dependently to 0.2, 0.4, and 0.8 of the control over the course of 6 min. As shown by the black line, the simulated data with the 0.4 of the control are quite similar to the changes in the baseline levels recorded actively with GSR.

Figure 5A shows the equivalent electrical model of human skin. The voltage and resistance of palmar and forearm skin in the model are described in E1 and E2, and R1 and R2, respectively. The skin surface and the extracellular tissue resistances between palmar and forearm skin show R and r, respectively. The R is known to be significantly larger than the r (Denda et al., 2001). On morphological evidence that the thickness of epidermis and the length of duct in the sweat gland in palm are larger and longer compared with these in forearm, we have hypothesized that E1 is larger than E2 because the concentrations of reabsorbed sodium and chloride ions at the extracellular tissues in palm may be higher than those in forearm. According to the evidence, the electrical current may flow from palm to forearm through the extracellular tissue space, resulting in flowing of electrical current from forearm to palm through the skin surface. Thus, the electrical current through the skin surface induces a voltage difference between palm and forearm skin, which is reflected GSR.

On the equivalent electrical model of human skin, when closing the eyes with stopping active palmar sweating, the reabsorbed sodium and chloride ions in the extracellular tissues of palmar skin return gradually to the blood stream, resulting in the time-dependent decrease in palmar voltage E1 in the model. When the palmar electrical voltage, E1, decreased to 0.8, 0.4, and 0.2 of the control values over the time course of 6 min, we simulated the changes in baseline GSR. The simulated data are demonstrated in Figure 5B. As shown by the black line, the data with the 0.4 value of control were quite similar to the changes in the baseline recorded actively with GSR in all human subjects with eyes closing.

### Self-Awareness of Drowsy in Human Subjects Decreased Gradually the Baseline GSR

**FIGURE 6:**
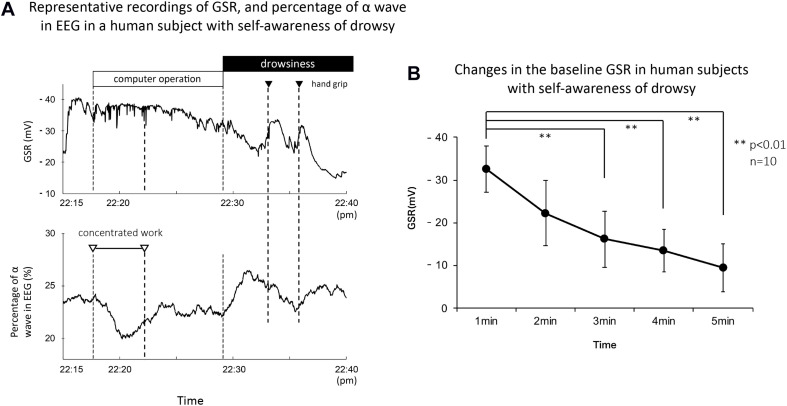
**(A)** Representative recordings of GSR, percentage of α wave in EEG, and R-R interval in ECG in a human subject with concentrated computer work and self-awareness of drowsy. The subject worked with a computer at mid-night between 22:15 and 22:40. He confirmed by himself the awareness with investigation of his recording data and the number of eyes closing and head down checked by the research assistant. The black column shows the period of self-awareness of drowsy confirmed by the subject. The white and black triangles demonstrate the period for his concentrated computer work and the trial of handgrip exercise to stay awake, respectively. **(B)** Summarized data for time course decreased the baseline GSR in human subjects with self-awareness of drowsy (*n* = 15). **p < 0.01.

Figure 6A shows representative recordings of GSR, percentage of α wave in EEG, and R-R interval of ECG in a human subject with self-awareness of drowsy. The subject worked with his computer at mid-night between 22:15 and 22:40. He first concentrated on his work, gradually became drowsy, and then tried two times the handgrip exercise to stay awake according to the suggestion of the research assistant. When he concentrated on the computer work, the GSR showed faster spikes with no or little change in the baseline GSR. Simultaneously, the rapid decrease in the percentage of α wave in EEG and tentative shorting of R-R interval in ECG were observed. Approximately 15 min after starting his work, the baseline GSR was gradually decreased and the percentage of α wave in EEG was simultaneously increased. Their responses agreed with his self-awareness of drowsy confirmed by himself. When he tried handgrip exercise to stay awake by oral suggestion of the research assistant, the baseline GSR was tentatively increased, and the percentage of α wave in EEG was slightly decreased. However, the second trial of handgrip exercise produced a tentative increase in the baseline GSR followed with a marked decrease. Similar tentative changes in the percentage of α wave in EEG were also observed. The self-awareness of drowsy-mediated decrease in the baseline GSR and increase in percentage of α wave in EEG were confirmed with all volunteers.

On the other hand, the changes in R-R interval of ECG were variable in the self-awareness of drowsy and the handgrip exercise to stay awake. Thus, when the volunteer confirmed the self-awareness of drowsy during 22:30 to 22:40, the R-R interval of ECG produced no or little change. When he tried handgrip exercise to stay awake at first trial, the R-R interval of ECG increased slightly. At the second trial, it decreased tentatively. Comparing with the changes in the baseline GSR and the percentage of α wave in EEG, the changes in R-R interval of ECG showed a marked variability.

Figure 6B demonstrates the summarized data for changes in baseline GSR in human subjects (*n* = 15) with self-awareness of drowsy. Around 3–4 min after starting self-awareness of drowsy in human subjects, the baseline GSR significantly decreased which is quite similar to the response obtained with eyes closing in the subjects (control 0 min, 36.7 ± 2.5 mV; 1 min, 26.8 ± 2.1 mV, *p* < 0.01 vs. 0 min; 2 min, 21.4 ± 1.8 mV, *p* < 0.01 vs. 0 min; 3 min, 18.3 ± 1.5 mV, *p* < 0.01 vs. 0 min; 4 min, 16.6 ± 1.3 mV, *p* < 0.01 vs. 0 min, *n* = 15).

## Discussion

In the present experiments, we demonstrated that (1) the handgrip exercise–mediated secretion of sweat in palmar sweating was observed at the top region of fingerprints in the thumb and the secreted sweat was quickly absorbed into the epidermis of palmar skin. (2) The appeared time of the sweat after starting maximal handgrip exercise was 1.43 ± 0.15 s (*n* = 30). The absorbed time of the sweat into the palmar skin was 6.02 ± 2.19 s (*n* = 30). (3) The faster phasic component of GSR with maximal handgrip exercise entirely agreed with the starting point of active palmar sweating and with decreasing point of the R-R interval in ECG in all volunteers. (4) Eyes closing in human subjects induced a significant time-dependent decrease in the baseline GSR; in contrast, eyes opening produced a significant increase in the baseline GSR in all subjects. (5) Eyes closing diminished active palmar sweating and increased the percentage of α wave in EEG in all subjects. (6) On the simulation with equivalent electrical model of human skin, when the palmar skin voltage in the model, E1, decreased time dependently to 0.4 of the control during 6 min, the simulated changes in the baseline GSR was completely consistent with the actual changes in baseline GSR recorded in human subjects with eyes closing. (7) The self-awareness drowsy during mid-night working induced a similar decrease in the baseline GSR and increase in the percentage of α wave in EEG to those observed with eyes closing in all subjects.

### Relationship Between the Phasic Component in GSR and Active Palmar Sweating

In the present experiment, the faster spike in GSR completely agreed with the starting point of active palmar sweating in all subjects. The correlation relationship between the GSR output and water loss in active palmar sweating was approximately linear. The relationship between the time delay in GSR and the corrected time delay in active palmar sweating was also approximately linear. The active sweating with high concentrations of Na^+^ and Cl^–^ ions in the sweat produces a decrease in the skin surface electrical resistance between palm and forearm skin, resulting in decrease in the skin surface electrical voltage difference between palm and forearm. This evidence may clearly explain the phasic component of GSR which changes to a downward direction in the GSR output. In addition, the phasic component of GSR correlated to water loss in active palmar sweating in human subjects (Figure 3B). Thus, the finding suggests that we are able to evaluate the strength of emotional stimulation in human subjects using the amount of phasic component in GSR.

In addition, the sweat secreted on the surface of palmar skin in human subjects with active palmar sweating was quickly absorbed into the epidermis of palm. The absorption of excreted sweat is a specific property of active palmar sweating, comparing with thermal sweating in the forearm, which may contribute to make the soft-cushion properties of palmar skin.

### Central Nervous System Contributed to Active Palmar Response and the Faster Phasic Component of GSR

On the changes in R-R interval of ECG, the maximum handgrip exercise decreased rapidly the R-R interval of ECG concomitant with changes in faster spike in GSR and active palmar sweating. In contrast, the self-awareness of drowsy produced no or little significant change in the R-R interval. The differences for the responses of R-R interval in ECG between handgrip exercise and aroused alertness may be related to the differences of contributed peripheral autonomic nervous activities between the exercise-mediated skin thermal perspiration and the drowsiness-mediated emotional palmar sweating. In addition, the changes in the R-R interval of ECG may be influenced by many other factors, i.e., the environmental stimulation included with light, the condition of vagal nerve activity, and a time-locked change in ECG. However, the detailed mechanisms of the variability will be needed in the future to investigate comprehensively.

In summary, these findings strongly suggest that active palmar sweating and the first spike in GSR are available tools for the evaluation of emotional reactions in human subjects (Darrow, 1927, 1937; Edelberg, 1977; Fowles, 1986; Denda et al., 2001: Boucsein, 2012).

### Eyes Closing and Opening in Human Subjects Produced Significant Changes in the Baseline GSR

Another important finding in the present study is that eyes closing in human subjects caused a significant decrease in the baseline GSR. The equivalent electrical model of human skin suggests that the eyes closing–mediated change in the baseline GSR may completely agree with the hypothesis that the palmar skin electrical voltage, E1, decreases time dependently to 0.4 of the control during 6 min. The decrease in the palmar skin electrical voltage may be explained by the morphological and physiological findings that residual sweat in the duct of the sweat glands is produced with eyes closing–mediated stopping active palmar sweating. Thus, Na^+^ and Cl^–^ ions and water within the duct of sweat glands are quickly reabsorbed into the extracellular tissues of palmar skin during stopping active sweating, and then time dependently moved into the blood stream. Changes in the transport of electrolytes and water may be related to decrease in palmar skin electrical voltage in the electrical model of human skin.

In addition, eyes closing simultaneously increased the percentage of α waves in EEG recordings. The changes may be concomitant with the findings that the amygdala and hippocampus in the CNS are responsible for active palmar sweating. In contrast, eyes opening induced active palmar sweating with increases in the input stimulation to the CNS center.

In support to the conclusion, Matsuo et al. (2015) have demonstrated using the perspiration ratemeter that eyelid opening in human subjects stretched mechanoreceptors in the supra-tarsal Müller muscle to activate the proprioceptive fibers supplied by the trigeminal mesencephalic nucleus. The strong trigeminal evocation activated cholinergic sudomotor nerve fibers innervated sweat glands and then induced active palmar sweating. This finding may agree with our conclusion that eyelid up movement–dependent eyes opening activates palmar sweating in human subjects (Figure 4A).

### The Self-Awareness Drowsy in Human Subjects Produced a Similar Decrease in the Baseline GSR to That Observed With Eyes Losing

The noteworthy finding in the present finding is that the self-awareness drowsy in human subjects at mid-night working with computer produced significantly a time-dependent decrease in the baseline GSR and increase in the percentage of α wave in EEG, the responses of which were quite similar to those obtained with eyes closing (Figure 6). The finding may be the first report in perspiration research. Thus, the finding will become a useful tool in the future to detect the clearness and drowsiness in human subjects. However, the present study is still preliminary and perspective. We should be needed in the future to clarify the usefulness with additional experiments.

## Conclusion

In conclusion, the faster phasic component in GSR completely agreed with the starting point of active palmar sweating. Changes in the baseline GSR in human subjects reflect closing or opening eyes. A similar decrease in the baseline GSR was observed with self-awareness of drowsy in human subjects. These changes will form the basis of useful tools for evaluating clearness and drowsiness in human subjects using GSR and active palmar sweating.

## Data Availability Statement

The raw data supporting the conclusions of this article will be made available by the authors, without undue reservation.

## Ethics Statement

The studies involving human participants were reviewed and approved by the ethical committee for human studies at Shinshu University School Medicine. The patients/participants provided their written informed consent to participate in this study.

## Author Contributions

All human experiments were performed in the laboratory of HM, NM, EI, SK, MS, and TO. Photomicrographs and videos were produced in the laboratory of HM, NM, EI, SK, and MS. TO: conception and design of the experiment. HM and TO: acquisition, analysis, and interpretation of data. TO: drafting the manuscript and critically revising it for intellectual content. All authors approved of the final version of the article and agree to be accountable for all aspects of the work to ensure that questions related to the accuracy or integrity of any part of the work are appropriately addressed. All persons designated as authors qualify for authorship, and all those who qualify for authorship are listed.

## Conflict of Interest

HM was employed by company SKINOS Co., Ltd. NM and EI were employed by company Rubycon Co., Ltd. SK was employed by company NEC Solution Innovators Co., Ltd. The remaining authors declare that the research was conducted in the absence of any commercial or financial relationships that could be construed as a potential conflict of interest.

## Abbreviations

GSR: galvanic skin response
ECG-: electrocardiograph
EEG: electroencephalograph
CNS: central nervous system
MRI-: magnetic resonance imaging.
